# Corroded iron stent increases fibrin deposition and promotes endothelialization after stenting

**DOI:** 10.1002/btm2.10469

**Published:** 2022-12-13

**Authors:** Yalan Deng, Yanbin Wen, Jun Yin, Jiabing Huang, Rongsen Zhang, Gui Zhang, Dongxu Qiu

**Affiliations:** ^1^ NHC Key Laboratory of Cancer Proteomics & Laboratory of Structural Biology, Xiangya Hospital Central South University Changsha Hunan People's Republic of China; ^2^ Department of Neurology, Xiangya Hospital Central South University Changsha Hunan People's Republic of China; ^3^ Department of Cardiology The Second Affiliated Hospital of Nanchang University Nanchang Jiangxi People's Republic of China; ^4^ Department of Ultrasonography, Second Xiangya Hospital Central South University Changsha China; ^5^ R&D Center, Lifetech Scientific (Shenzhen) Co Ltd Shenzhen People's Republic of China

**Keywords:** corrosion, endothelial cell, endothelialization, fibrin deposition, iron stent

## Abstract

Poststent restenosis is caused by insufficient endothelialization and is one of the most serious clinical complications of stenting. We observed a rapid endothelialization rate and increased fibrin deposition on the surfaces of the corroded iron stents. Thus, we hypothesized that corroded iron stents would promote endothelialization by increasing fibrin deposition on rough surfaces. To verify this hypothesis, we conducted an arteriovenous shunt experiment to analyze fibrin deposition in the corroded iron stents. We implanted a corroded iron stent in both the carotid and iliac artery bifurcations to elucidate the effects of fibrin deposition on endothelialization. Co‐culture experiments were conducted under dynamic flow conditions to explore the relationship between fibrin deposition and rapid endothelialization. Our findings indicate that, from the generation of corrosion pits, the surface of the corroded iron stent was rough, and numerous fibrils were deposited in the corroded iron stent. Fibrin deposition in corroded iron stents facilitates endothelial cell adhesion and proliferation, which, in turn, promotes endothelialization after stenting. Our study is the first to elucidate the role of iron stent corrosion in endothelialization, pointing to a new direction for preventing clinical complications caused by insufficient endothelialization.

## 1 INTRODUCTION

A stent implantation is widely used to treat coronary stenosis.^1^, ^2^ However, intimal injury during implantation surgery is inevitable because of the mechanical damage caused by the surgery.^3^, ^4^ In addition, intravascular stent implantation causes a persistent prothrombotic milieu until the endothelial cells fully cover the stent struts.^5^ Endothelial monolayers not only serve as a dynamic physiological border to isolate vascular surrounding tissue from circulating blood flow but also provide a nonadhesive surface for leukocytes and platelets.^6^ Moreover, endothelial monolayers have been shown to regulate platelet activation and aggregation, which are closely correlated with occlusive thrombosis.^7^ Clinicians usually recommend dual antiplatelet therapy to prevent poststent restenosis caused by insufficient endothelialization. However, patients are required to undergo dual antiplatelet therapy for up to 12 months until the endothelial monolayer is fully formed. Unfortunately, antiplatelet agents, such as aspirin and clopidogrel, pose a high risk of life‐threatening hemorrhage.^8^, ^9^ Insufficient endothelial monolayer recovery is the primary cause of long‐term complications after stenting.^10^, ^11^, ^12^ Thus, promoting endothelialization not only reduced the incidence of poststent restenosis but also decreased the antiplatelet drug therapy period. Although researchers have struggled to increase endothelial cell coverage, insufficient endothelialization remains one of the most serious clinical complications impeding interventional surgery development. Novel investigations on the promotion of endothelial cell coverage after stenting are urgently required. Currently, numerous biological experiments have been performed to evaluate the effect of device surface chemistry on the surrounding microstructure of blood‐contacting materials as well as determine the effectiveness of various types of coating strategies in enhancing endothelialization and suppressing vascular smooth muscle cells (VSMCs) proliferation. Qing et al. demonstrated that a catalytic surface improved endothelial cell compatibility but inhibited VSMC proliferation by depositing Ti‐Cu coatings on stainless steel substrates.^13^ Ming et al. provided evidence that fabricated shellac coatings decrease neointimal hyperplasia by regulating VSMC phenotypic transformation. This coating design also exhibited excellent blood compatibility and antibacterial activity.^14^ In addition, vascular endothelial growth factor (VEGF) and anti‐CD34 antibodies were immobilized onto the surface of the Ni‐Ti alloy sheet, which improved endothelial cell proliferation.^15^ Similarly, the endothelialization process was promoted by conjugating human blood exosomes onto the surface of a polydopamine‐coated 316L stainless steel stent.^16^ Carbon coatings deposited using a mixed‐mode high power impulse magnetron sputtering (HiPIMS) process provide biocompatible interfaces suitable for blood‐contacting devices.^17^ Moreover, optimized Ti‐xCu coatings with Ti and Cu/CuTix crystals improved endothelial cell compatibility and inhibited VSMC proliferation.^18^ In contrast to modifying surface coatings or biological coating surfaces, special attention has also been paid to drug‐loaded coatings. By encapsulating paclitaxel‐loaded mesoporous silica nanoparticles within electrospun polylactic acid fibers, bio‐functional stents covered with dual drug‐loaded electrospun fibers achieved programmed VEGF and paclitaxel release, which increased endothelialization and reduced long‐term stenosis.^19^ Additionally, the poly‐dopamine/hexanediamine‐chondroitin sulfate C coating exhibited rapid endothelialization and better blood compatibility in cardiovascular implants.^20^

Our previous study observed rapid endothelialization after the implantation of a nitrided iron stent. Thus, we speculated that the rough and uneven surface of the corroded iron stent influences the adhesion of blood components and promotes endothelialization. To verify this hypothesis, we performed corrosion experiments to confirm the degradation characteristics of the iron stent after implantation and detailed investigations of the morphology of the corrosion pits generated by iron stent degradation. An experimental arteriovenous shunt model was introduced to determine blood component adhesion. We found that owing to the generation of corrosion pits, the surface of the iron stent became rough and uneven, which increased fibrin deposition on the corroded iron stent surface. To further evaluate the effects of fibrin deposition on endothelialization, we implanted a corroded iron stent in both the carotid and iliac artery bifurcations. The results showed that corroded iron stents increased fibrin deposition and promoted nonendothelial formation. To explore the effects of fibrin deposition on endothelialization, we conducted co‐culture experiments under dynamic flow conditions. These results indicated that fibrin deposition in the corroded stent enhanced endothelialization by stimulating endothelial cell adhesion and proliferation. Our study as a whole is the first to delineate the role of iron stent corrosion in endothelialization, pointing to a new direction for enhancing endothelialization after stenting.

## 2 RESULTS

### 2.1 Corrosion pits were extensively generated in the corroded iron stent

**FIGURE 1:**
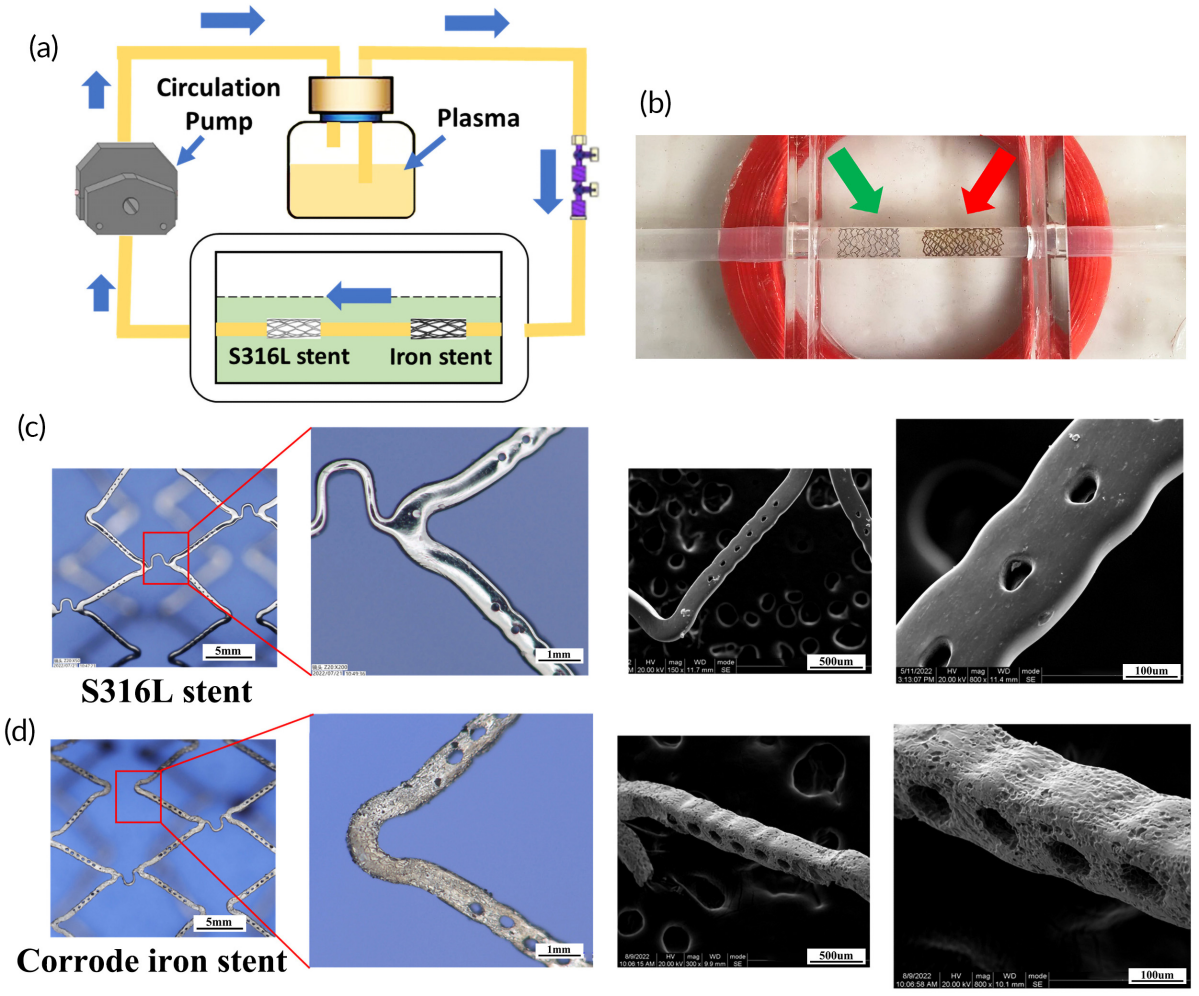
Corrosion pits generated in the surface of the iron stent after degradation. (a) Schematic diagram of the dynamic circulation device: a peristaltic pump was introduced to mimic vessel pulsation, the silastic tube was connected to the peristaltic pump, and the pump continuously transferred plasma from one end to the other. The iron stent was deployed into a transparent conduit. Additionally, a S316L stent was placed near the iron stent as a control. (b) As the circulation time increased, the appearance of the iron stent turns dark, and black iron corrosion granules were generated around the struts. The red and green arrows indicate the S316L stent and iron stent, respectively. (c, d) Pitting corrosion was extensively generated in the corroded iron stent, and the surface became rough and uneven, whereas no corrosion pits were observed in the S316L stent, and the surface remained polished.

A circulation device was introduced to delineate the corrosion of the iron stent, as shown in Figure 1a. The stent was deployed in the middle of a transparent conduit, and plasma was circulated through it. Additionally, a 316L stainless steel stent (S316L stent) was placed near the iron stent as a control. As shown in Figure 1b, yellow iron corrosion granules were observed around the stent strut. As the experimental time increased, the iron stent became surrounded by corrosion granules. Moreover, corrosion pits were generated in the iron stent, leading to a rough and uneven surface (Figure 1c,d). In contrast, few corrosion granules were observed in the S316L stent, and its surface remained polished. These results demonstrate the surface changes in the corroded iron stent, confirming the degradation characteristics of the iron stent following implantation.

### 2.2 Numerous fibrins were deposited on the corroded iron stent surface

**FIGURE 2:**
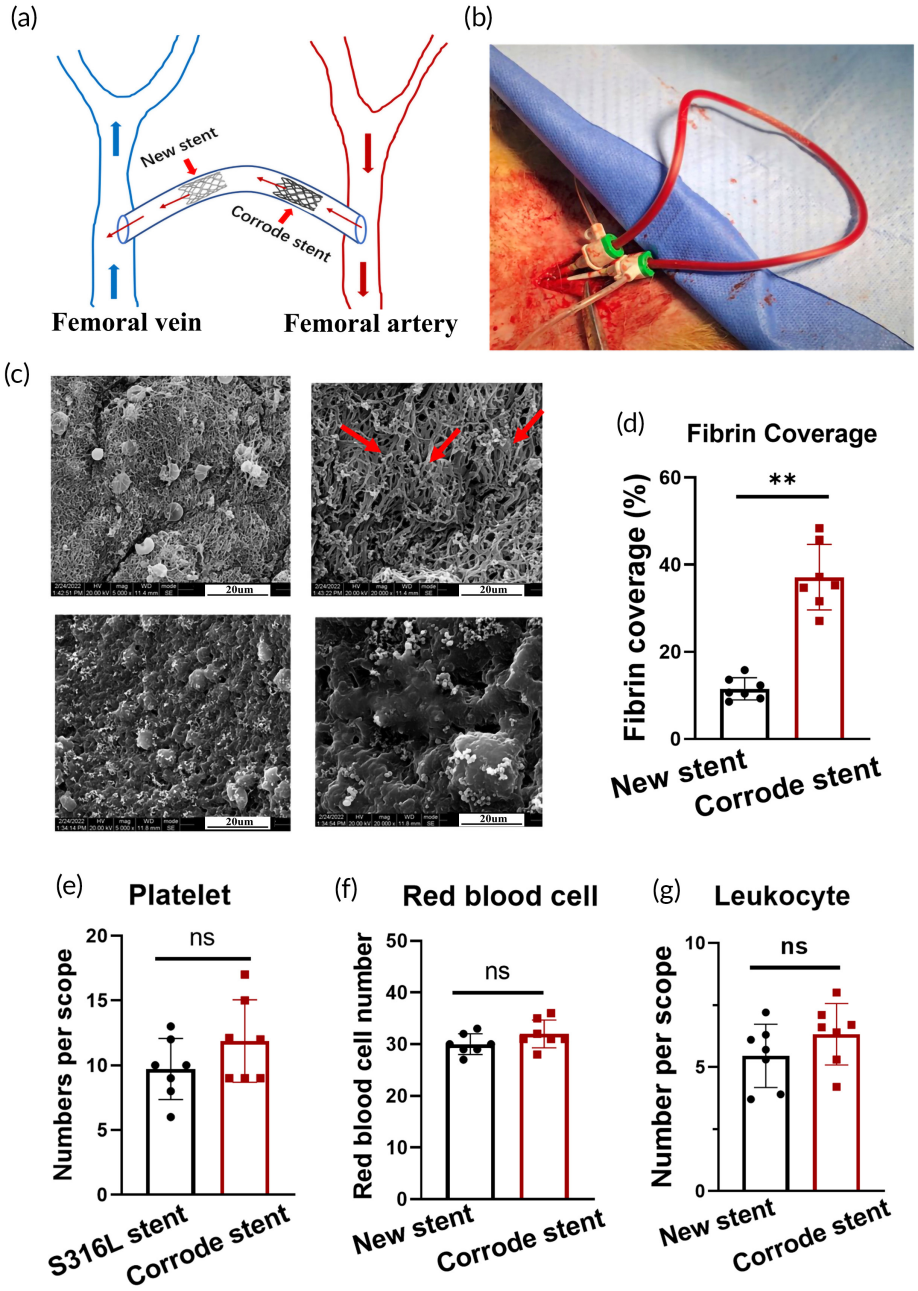
Numerous fibrins deposited on the corroded iron stent surface. (a, b) Schematic diagram of the arteriovenous shunt experiment. A transparent conduit was connected between the femoral and vein arteries in rabbits. The corroded iron stent was deployed in the middle of the conduit. A new iron stent was inserted as the control. (c, d) Numerous fibrins are deposited uniformly over the entire surface of the corroded iron stent. These fibrin networks become increasingly dense with prolonged circulation time. However, considerably less fibrin is present in the control stent. The red arrow indicates the fibrin in the corroded iron stent. (e) No statistically significant differences were identified regarding the platelet, red blood cell, or leukocyte levels between the two stents. Bars represent the mean ± standard deviation (SD). *p < 0.05; “ns” represents no statistical difference.

As the surface of the corroded iron stent became rough and uneven, we speculated that it may influence the adhesion of blood components, such as erythrocytes, platelets, leukocytes, and fibrin. An arteriovenous shunt experiment was performed to address this issue, where a transparent conduit was connected to the femoral artery and vein in the rabbit model. A corroded iron stent was then deployed in the middle of the conduit. An iron stent without corrosion was used as a control. A schematic of this process is presented in Figure 2a,b. With prolonged experimental time, we found numerous fibrin strands deposited uniformly over the entire surface of the corroded iron stent, forming thick fibrous networks in some areas. However, the surface of the new iron stent was relatively clean, with little fibrin deposition on its surface (Figure 2c,d). In contrast to successful fibrin deposition, no statistical differences were found in the adhesion of red blood cells, platelets, and leukocytes between the two groups (Figure 2e–g).

### 2.3 Fibrin on corroded iron stent promotes endothelialization

**FIGURE 3:**
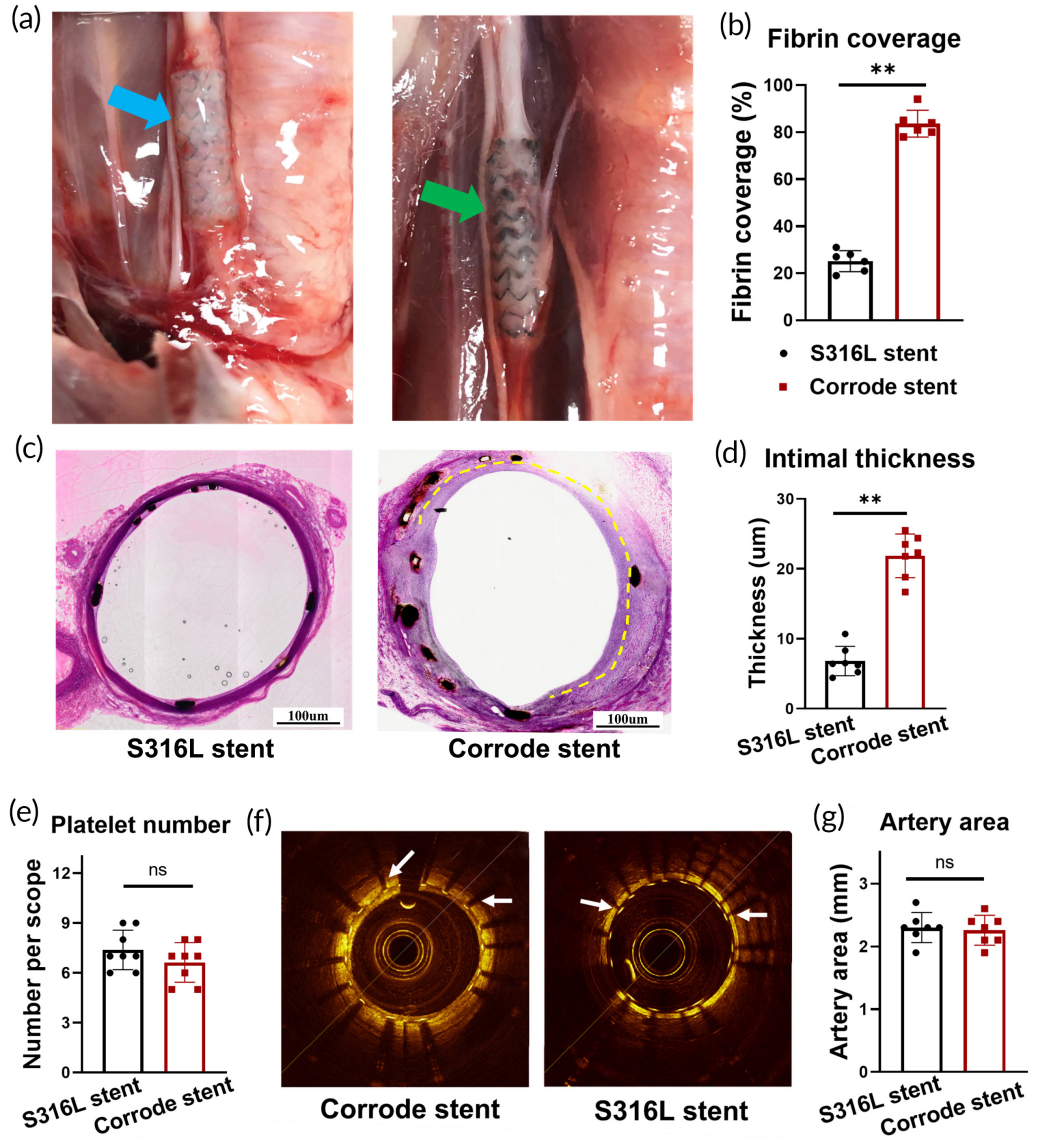
Fibrin deposition in corroded iron stents promotes endothelialization. (a) A corroded iron stent was implanted into the carotid arteries of rabbits, and the S316L stent was deployed as the control. The blue arrow indicates the S316L stent, and the green arrow indicates the corroded iron stent. (b) Fibrin covers most parts of the corroded iron stent surface (Day 15). (c, d) Rapid endothelialization in the corroded iron stent by Day 45. Considerably less endothelialization is present in the S316L stent. (e) Low platelet accumulation in the corroded iron stent surface. (f) Optical coherence tomography (OCT) indicates the transluminal area of the implanted site was smooth with no occlusive thrombosis formed. The white arrows indicate the stent struts embedded in the surrounding artery tissue. (g) No differences were found in the lumen cross‐sectional area between the corroded iron stent and the control. Bars represent the mean ± standard deviation (SD). *p < 0.05; **p < 0.01; “ns” represents no statistical difference.

Fibrin deposition promotes endothelial cell attachment, spreading, and proliferation. Results of the arteriovenous shunt experiment indicated an increase in fibrin deposition in the corroded iron stents. Therefore, we explored the role of fibrin deposition in endothelialization in vivo. The corroded iron stent was implanted into the carotid artery in a rabbit model by performing endovascular surgery, and the S316L stent was deployed as a control (Figure 3a). All rabbits (*n* = 14; weight, 5.0 kg ± 0.4 kg; 7 males and 7 females) were successfully stented, and no peri‐procedural complications occurred. Days 15 and 45 were set as experimental time points. All the rabbits remained healthy until the final follow‐up period. Fibrin covered the corroded stent surface on Day 15 (Figure 3b). With prolonged implantation time (on Day 30), an endothelial monolayer was observed on the corroded iron stent surface (Figure 3c,d). In accordance with the regeneration of endothelial monolayer, the expression of CD31, a surface marker of endothelial cell, was also increased in corroded iron stent group (Figure S1a,b). However, little fibrin deposition and few endothelial monolayers were observed in the S316L stent.

Although fibrin deposition promotes endothelial monolayer coverage, it binds with high specificity to integrin receptors on platelets and activates platelet aggregation. Therefore, we determined the adhesion of platelets to the corroded stent surface. Our results indicated a small number of platelets accumulated on the corroded iron stent surface. However, no statistical differences were identified between the groups (Figure 3e). Moreover, optical coherence tomography (OCT) observation indicated that the transluminal area of the implantation was smooth with no occlusive thrombosis (Figure 3f,g).

**FIGURE 4:**
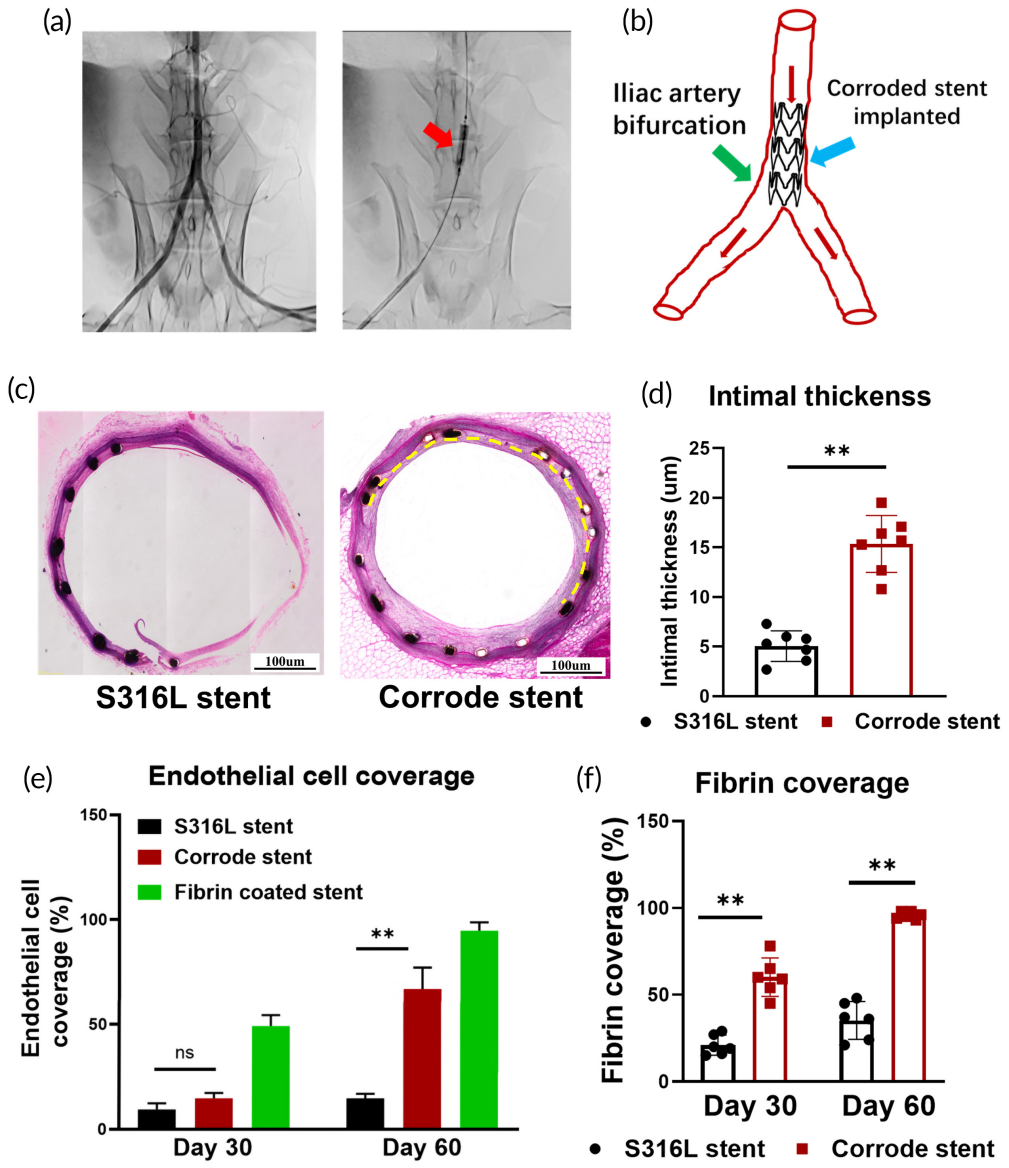
A corroded iron stent promotes endothelialization at artery bifurcation sites. (a) Artery angiography during stent implantation in the iliac artery bifurcation. The red arrow indicates the iliac artery bifurcation. (b) Schematic diagram of iliac artery bifurcation implantation experiment. (c) Complete endothelial cell coverage in the corroded iron stent. However, the struts were exposed to the vascular lumen, and the endothelialization process was delayed in the S316L stent. (d) Endothelial monolayer thickness is higher on the corroded iron stent. (e) Rapid endothelialization process on the corroded stent surface. However, much less endothelial cell coverage is found in the S316L stent. (f) Fibrin deposition is increased in the corroded iron stent. Bars represent the mean ± standard deviation (SD). *p < 0.05; **p < 0.01

Clinical observations have indicated that the rate of endothelialization is delayed when the stent is implanted at the artery bifurcation. This is due to the dramatic change in the direction of blood flow in the artery bifurcations, leading to a much higher shear stress on the vessel wall and insufficient endothelialization.^21^, ^22^ To further address the role of fibrin deposition in artery bifurcation endothelialization, we implanted a corroded iron stent in the iliac artery bifurcation (Figure 4a,b). The fibrin‐coated iron stent and the S316L stent were used as controls. All rabbits (*n* = 14; weight, 5.2 kg ± 0.5 kg; 7 males and 7 females) were successfully stented, and no periprocedural complications occurred during the observation period. Days 30 and 60 were set as time points. Remarkably, numerous fibrin deposits were observed on the surface of the corroded iron stent. After prolonged implantation, complete endothelial cell coverage was observed on the corroded iron stent surface (Day 60). However, considerably less fibrin was found on the S316L stent, and its surface was smooth with little endothelial cell coverage (Figure 4c–f). Thus, we confirmed that fibrin deposition in a corroded iron stent promoted endothelialization in both carotid artery and iliac bifurcation implantation models.

### 2.4 Fibrin deposition facilitated endothelial cell adhesion and proliferation

**FIGURE 5:**
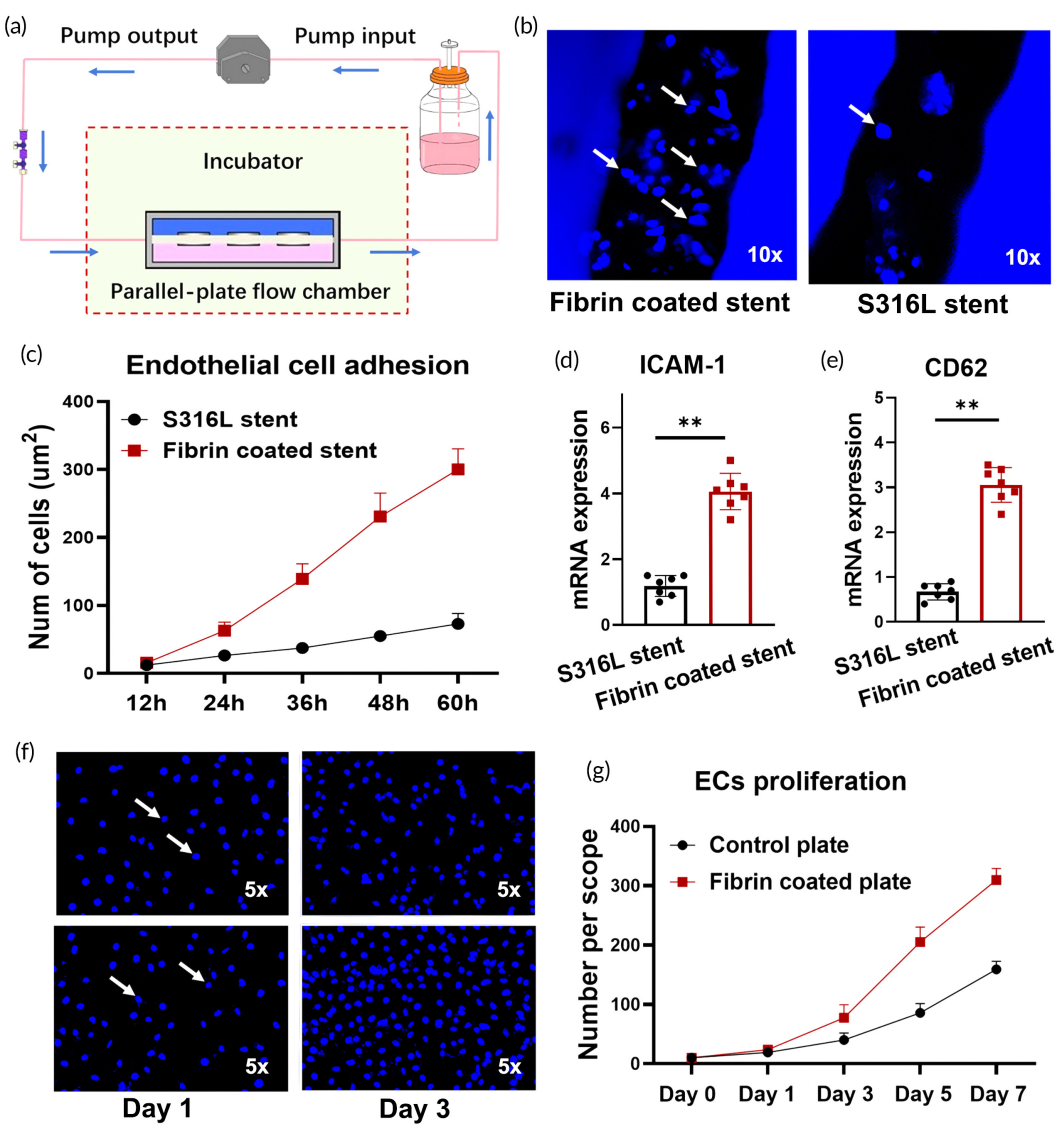
Fibrin deposition promotes endothelial cell adhesion and proliferation. (a) Schematic of the parallel‐plate flow chamber system. Endothelial cells were seeded on both surfaces of the fibrin‐coated iron stent and the new iron stent (control group). The chamber was presheared under low shear stress. Endothelial cells were subjected to high shear, and the remaining cells were analyzed. (b, c) Higher endothelial cell density on the surface of the fibrin‐coated iron stent. The white arrow indicates the endothelial cells attached to the stent surface. (d, e) Intercellular adhesion molecule 1 (ICAM‐1) and the leukocyte receptor CD62 were up‐regulated in the fibrin‐coated stent. (f, g) Increased number of endothelial cells in the fibrin‐coated plate group. The white arrow indicates the endothelial cells stained by 4′,6‐diamidino‐2‐phenylindole (DAPI). Bars represent the mean ± standard deviation (SD). *p < 0.05; **v < 0.01; “ns” represents no statistical difference.

Endothelial cell adhesion to the stent surface is the initial step in endothelialization. Endothelialization increased following fibrin deposition in the corroded iron stent. We speculated that fibrin deposition promotes endothelialization by increasing endothelial cell adhesion. Therefore, the relationship between fibrin deposition and endothelial cell adhesion was investigated in vitro. Notably, endothelial cell adhesion responds to fluid flow. Therefore, the biological response of endothelial cell adhesion must be examined under dynamic flow conditions, and a parallel‐plate flow chamber system was introduced to answer this question. Endothelial cells were allowed to attach for 30 min on the surface of both the fibrin‐coated iron stent and the S316L stent (control group). The chamber was then presheared with low shear stress (0.5 dynes/cm^2^). Following preconditioning, the endothelial cells were subjected to high shear stress (2 dynes/cm^2^) to induce cell detachment, and the remaining cells were analyzed. A schematic of the device is shown in Figure 5a. Although no significant differences were detected in the number of adhered endothelial cells during the initial observation period (12 h), we found an increased number of endothelial cells attached to the fibrin‐coated stent after 24 h (Figure 5b). Consistent with cell adhesion, noticeable changes were observed in cell adhesion‐related mRNA levels. We found that endothelial cells showed increased ICAM‐1 and CD62 mRNA expression in the fibrin‐coated stent group compared to the S316L stent group (Figure 5d,e).

In addition to endothelial cell adhesion, endothelial cell proliferation plays an essential role in endothelialization. The co‐culture medium was used to quantitatively assess endothelial cell proliferation after fibrin deposition. A fibrin‐coated plate and a nontreated plate (negative control) were included. Endothelial cells were labeled with 4′,6‐diamidino‐2‐phenylindole (DAPI). Although no statistically significant difference was observed in the number of endothelial cells on Day 1, many cells were observed on the surface of the fibrin‐coated plate on Day 3, and the number of endothelial cells on the coated plate was approximately 200% higher than that on the untreated plate (Figure 5f,g). Consistent with the results of the co‐culture experiment, the CCK‐8 assay indicated that the absorbance value at 450 nm increased in the fibrin‐coated plate group (Figure S1c). Moreover, the percentage of endothelial cells in the S phase was much higher than that in the untreated plate group (Figure S1d). These results suggest that fibrin deposition promotes endothelialization by facilitating endothelial cell adhesion and proliferation.

### 2.5 Fibrin deposition promotes endothelialization via VEGF‐A upregulation

**FIGURE 6:**
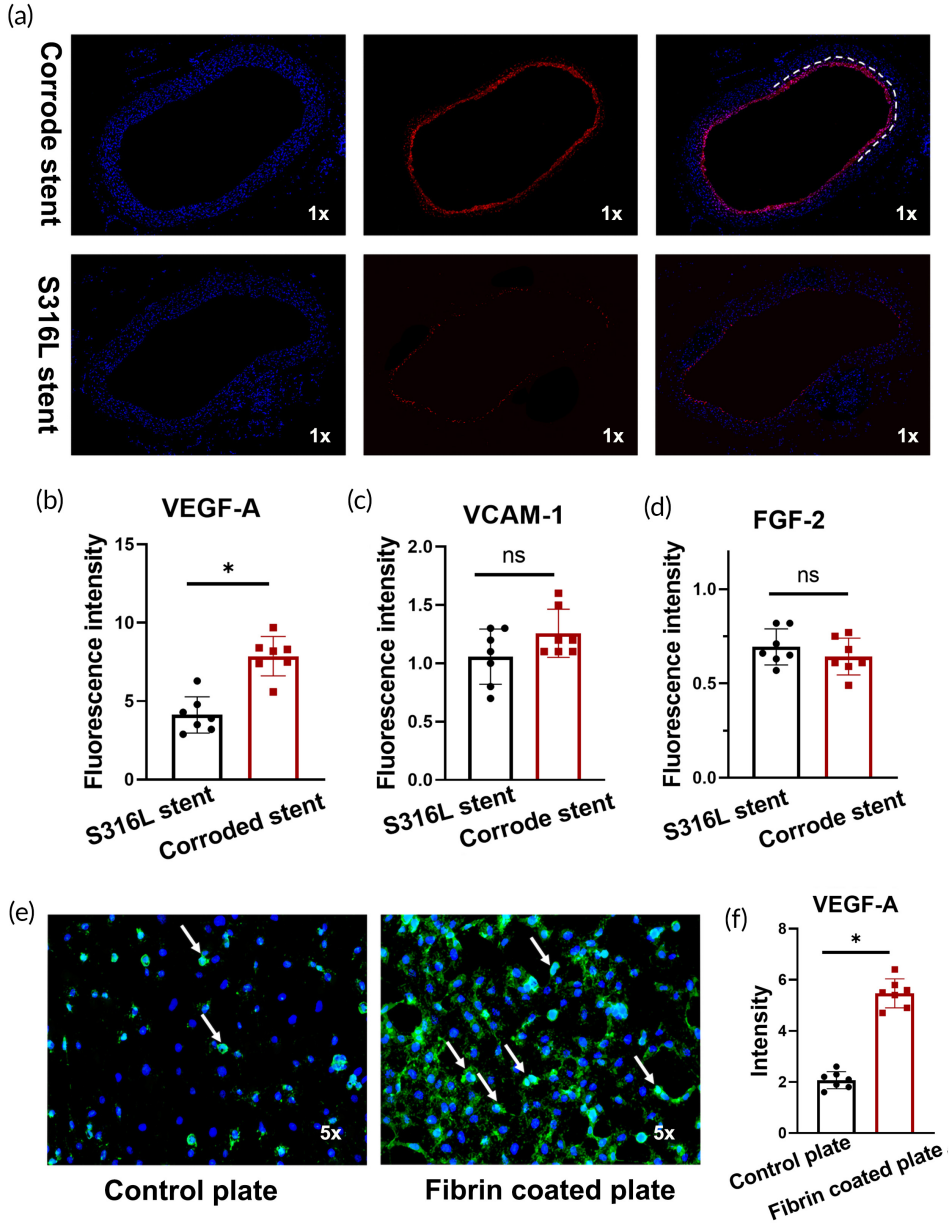
Corroded stent surfaces promote endothelialization via VEGF‐A upregulation. (a, b) The intensity value of VEGF‐A is elevated in the corroded iron stent. The white arrow indicates the VEGF‐A‐positive endothelial cells. (c, d) No statistically significant differences were measured in the expression of VCAM‐1 and FGF‐2 between the groups. (e, f) Endothelial cell exposure to a fibrin‐coated plate increased VEGF‐A expression. Consistently, the number of endothelial cells is also elevated in the fibrin‐coated plate group. The white arrow indicates the positive signal of VEGF‐A staining. Bars represent the mean ± standard deviation (SD). *p < 0.05; **p < 0.01; “ns” represents no statistical difference.

VEGF‐A is a strong endothelial cell activator. The endothelialization process increased in the corroded iron stent, which led us to further explore the role of VEGF‐A in endothelialization. We first measured VEGF‐A expression in artery tissues implanted with corroded iron stents. Coinciding with rapid endothelialization, VEGF‐A displayed high‐intensity values in corroded iron stent artery tissue, indicating elevated expression of VEGF‐A induced by fiber adhesion (Figure 6a,b). In addition to VEGF‐A, other molecules that could potentially influence endothelial cell growth, including VCAM‐1 and FGF‐2, were examined. However, these molecules were not induced after the implantation of the corroded iron stent (Figure 6c,d). To further study the effects of VEGF‐A on endothelialization, we performed an ex vivo experiment using a co‐culture medium. A fibrin‐coated plate and an untreated plate (negative control) were used. As shown in Figure 6e,f, endothelial cell exposure in the fibrin‐coated plate increased VEGF‐A expression. In accordance with VEGF‐A, the number of endothelial cells in the fibrin‐coated plate was higher than that in the control (Figure S1e,f). To confirm this finding, we disabled the VEGF‐A function by applying an anti‐VEGF‐A antibody. As expected, blocking the expression of VEGF‐A significantly reduced the number of endothelial cells on fibrin‐coated plates. In contrast, rabbit immunoglobulins (used as negative controls) showed little effect on endothelial cells (Figure S1g,h).

### 2.6 Hemocompatibility evaluation following corroded iron stent implantation

**FIGURE 7:**
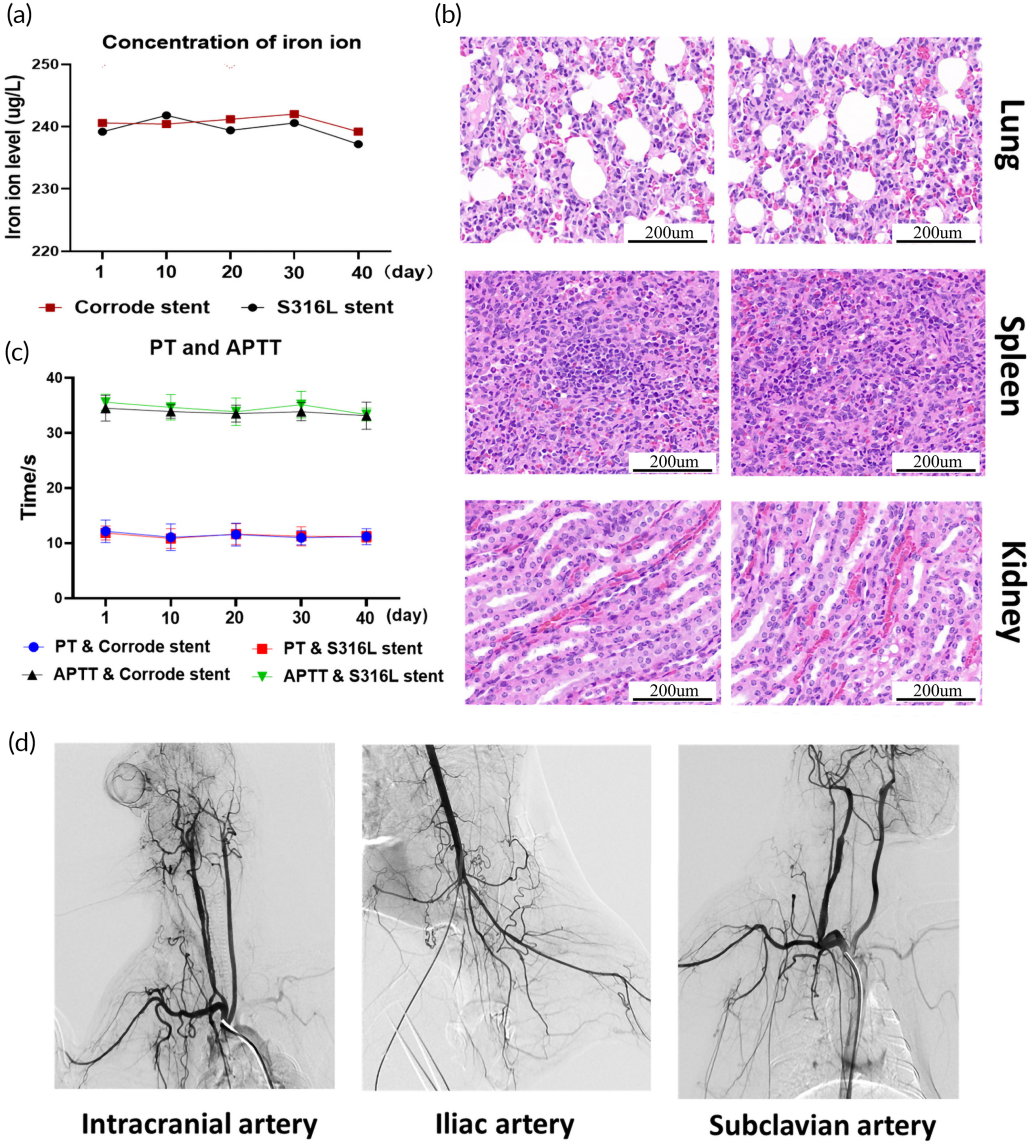
Hemocompatibility evaluation after the corroded iron stent is implanted. (a) The serum iron ion concentration is normal at different time points (Days 1, 10, 20, 30, and 40) after corroded iron stent implantation. (b) No abnormalities are present in the histomorphology observations of the spleen, kidney, and lungs. (c) The prothrombin time (PT) and activated partial thromboplastin time (APTT) were similar to those in the S316L stent at different time points (Days 1, 10, 20, 30, and 40). (d) No notable occlusion is observed in the distal branch of the iliac, intracranial, or subclavian arteries. Bars represent the mean ± standard deviation (SD); “ns” represents no statistical difference.

Hemocompatibility is a basic requirement of biomedical materials. Therefore, it is essential to evaluate the hemocompatibility of corroded iron stents following implantation. During corroded iron stent degradation, iron ions are released from the stent and high concentrations of iron ions can cause heavy metal intoxication. Thus, iron ions must be dynamically monitored after implantation. As shown in Figure 7a, the concentration of serum iron ions was normal at different time points (Days 1, 10, 20, 30, and 40). Additionally, iron ions may accumulate in vital organs and damage organ function over time. Therefore, we examined the histomorphology of the spleen, kidneys, and lungs. No abnormalities were observed in these organs (Figure 7b). The surface of the corroded iron stent was rough and uneven, which may have triggered the activation of the coagulation factors. Thus, the prothrombin time (PT) and activated partial thromboplastin time (APTT) tests were performed after deploying the corroded iron stent. As shown in Figure 7c, despite the extensive corrosion pits generated on the corroded iron stent surface, PT and APTT were similar to those of the S316L stent. Corrosion granules were produced from the iron stent during degradation. Corrosion granules may be transported by blood flow and block downstream distal branches. Therefore, the risk of vessel embolism after corroded iron stent implantation must be evaluated, and follow‐up angiography was conducted. As presented in Figure 7d, the peripheral blood vessels in the intracranial, iliac, and subclavian arteries were clearly visualized, indicating that no embolism had formed in the distal branch.

## 3 DISCUSSION

According to a research report from the World Health Organization, the morbidity and mortality rates of cardiovascular diseases (CVDs) are much higher than those of other disease types.^23^ Currently, interventional surgery is the most effective treatment for acute CVDs,^24^ wherein percutaneous transvascular balloon angioplasty and stent implantation are commonly used.^2^ However, more than 30% of patients suffer from obstruction of the treated artery within 3 months, owing to insufficient endothelial cell recovery.^3^, ^4^ The endothelial monolayer not only functions as a dynamic physiological border isolating the surrounding tissue from circulating blood flow, but it also provides a nonadhesive surface for leukocytes and platelets in the vascular system.^25^, ^26^ Moreover, endothelial monolayers regulate platelet activation and aggregation, which are closely associated with occlusive thrombosis after interventional procedures.^6^, ^7^ Therefore, promoting neointimal coverage and restoring endothelial function have emerged as crucial concerns after stent implantation surgery.

Bioresorbable stents are heralded as the fourth revolution in interventional technology and are designed to avoid the long‐term health risks posed by drug‐eluting stents.^27^, ^28^ Iron and iron alloys are candidate materials for bioresorbable stents, owing to their excellent mechanical performance and biocompatibility.^29^, ^30^, ^31^ Furthermore, we recently observed rapid endothelialization after iron stent corrosion. With the degradation of the iron stent, numerous corrosion pits are generated on its surface.^32^ Therefore, we speculated that with the production of corrosion pits, the surface of the iron stent became rough and uneven, thereby promoting the adhesion of blood components, which ultimately influenced endothelial cell coverage. To verify this, we first described the morphology of the corrosion pit by deploying an iron stent in a plasma‐circulation device. Because of the excellent corrosion resistance of the 316L stainless steel stent (S316L sent), we introduced the S316L stent and set it as the control to elucidate the corrosion properties of the iron stent. As expected, yellow corrosion granules were extensively generated around the stents. Moreover, numerous corrosion pits were generated around the stent, with corrosion granules carried away by the flowing fluid, and the surface became rough and uneven. These findings describe the development of corrosion pits and confirm the degradation characteristics of iron stents after implantation.

Biomaterial properties such as surface roughness and composition have been shown to influence their interaction with surrounding blood proteins.^33^ Although the surface changes of corroded iron stents are described above, the effects of corrosion pits on the adhesion of blood components need to be further elucidated. We performed an arteriovenous shunt experiment using a rabbit model and deployed the corroded iron stent in a transparent conduit simultaneously connected to the femoral artery and vein. A new iron stent was used as a control. Notably, we found that short, thin fibrin strands developed uniformly over the entire surface of the corroded stent, forming thick fibrous networks in some areas. Moreover, these fibrin networks grew and became increasingly dense with prolonged circulation time. In contrast, the surface of the new iron stent was relatively clean, with little fibrin being deposited on the surface. In addition to changes in fibrin attachment, a rough surface may also influence the adhesion of other blood components, such as platelets, red blood cells, and leukocytes. Therefore, we measured the adhesion between the components. However, no differences in blood components were identified between the two stents. Hence, we concluded that iron stent corrosion increased fibrin deposition on its surface.

Fibrin is a large, complex, fibrous glycoprotein with three pairs of polypeptide chains linked by 29 disulfide bonds.^34^, ^35^ Fibrin deposition is essential for hemostasis and angiogenesis. In particular, fibrin enhances the cellular production of extracellular matrix proteins to serve as a basement membrane, which is essential for achieving stable microvascular networks.^35^, ^36^ Therefore, temporary fibrin deposition promotes cell attachment, spreading, migration, and alignment.^37^, ^38^ Our previous findings suggest increased endothelialization after iron stent corrosion. Thus, we speculated that rapid endothelialization was involved in fibrin deposition on the corroded iron stent surface. A convincing and novel arterial implantation model was used to test this hypothesis. Clinical observations indicated that the endothelialization process was dramatically delayed if the stent was deployed at the artery bifurcation. The dramatic change in the direction of blood flow at the artery bifurcation and the accompanying shear stress were much higher on the vessel wall in this position, impeding endothelialization. To address the effects of fibrin deposition on endothelialization, we chose the iliac artery bifurcation and implanted a corroded stent in this position. Additionally, the fibrin‐coated iron stent and S316L stent were used as controls. Remarkably, rapid endothelialization was observed in fibrin‐coated stents during the early experimental period. With a prolonged implantation time, more fibrin deposits were detected on the surface of the corroded iron stent. Subsequently, neointimal coverage of the corroded stent is initiated. However, considerably less fibrin deposition was observed in the S316L stent, and its surface was smooth with little endothelial cell coverage. In short, by introducing the bifurcation artery implantation model, we demonstrated that iron stent corrosion promoted fibrin deposition, thereby increasing the endothelialization rate.

Fibrin serves as an extracellular matrix protein that provides the basis for endothelial cell adhesion and growth.^37^, ^38^ Fibrin coating of polytetrafluoroethylene prostheses increased endothelialization.^39^ Moreover, fibrin coating supports endothelial cell maturation and stabilization under blood‐flow conditions.^40^, ^41^ Indeed, fibrin can bind to a variety of blood proteins, including fibronectin, von Willebrand factor, albumin, and VEGF. Fibrin participates in cell growth, thrombosis, and inflammation.^42^, ^43^ Endothelialization was promoted after fibrin deposition on the corroded stent surface. Therefore, we propose that fibrin attachment enhances endothelialization by increasing endothelial cell adhesion. To test this hypothesis, we performed novel ex vivo experiments by introducing fibrin‐coated stents. In addition, to mimic the real circumstances of blood flow, the co‐culture medium was subjected to dynamic flow conditions, and a parallel‐plate flow chamber system was used. As expected, we observed faster endothelial cell adhesion on the fibrin‐coated stent surfaces. Moreover, noticeable changes in cell adhesion‐related mRNA levels were observed with the stent. In addition to endothelial cell adhesion, cell proliferation was measured quantitatively. However, no significant differences were observed during the early experimental period. In contrast, the number of cells began to increase in the fibrin‐coated plate with prolonged incubation time, and the number of endothelial cells was approximately 200% higher in the fibrin‐coated plate than in the control plate. Based on these results, we demonstrated that fibrin deposition enhanced endothelialization by promoting endothelial cell adhesion and proliferation.

Endothelialization is a complex process that involves endothelial cell adhesion and proliferation. VEGF‐A promotes endothelial cell growth via both direct and indirect mechanisms. VEGF‐A stimulates endothelial cell migration and proliferation near microvasculature.^44^, ^45^, ^46^ Therefore, VEGF‐A is recognized as an important endothelialization factor. As a rapid endothelialization process was observed in the fibrin‐coated stent, we further determined the role of VEGF‐A in endothelialization. We detected VEGF‐A expression in a fibrin‐coated stent in vivo using immunofluorescence (IF) staining. Consistent with rapid endothelial cell coverage, VEGF‐A IF results displayed high‐intensity values in the fibrin‐coated stent group. Furthermore, an ex vivo experiment was conducted, and an anti‐VEGF‐A antibody was used to confirm this finding by inhibiting VEGF‐A function. As expected, VEGF‐A inhibition dramatically reduced the number of endothelial cells on the surface of fibrin‐coated stents. In contrast, control rabbit immunoglobulin did not have a significant effect on the endothelial cell number. These findings provide evidence that fibrin deposition promotes endothelialization in corroded iron stents via the upregulation of VEGF‐A.

## 4 CONCLUSIONS

The promotion of endothelial cell coverage and the restoration of endothelial function after stenting remain major concerns. Researchers worldwide have struggled to increase endothelial cell coverage. Additionally, poststent restenosis caused by insufficient endothelialization remains a serious clinical complication following stent implantation. Our study highlights the role of iron stent corrosion in the promotion of endothelialization. First, we delineated the morphology of the corrosion pits and confirmed the degradation characteristics of iron stents after implantation. Moreover, by implanting a corroded iron stent in both the carotid and iliac artery bifurcations, we determined that the corroded iron stents increased fibrin deposition and promoted endothelialization. To elucidate the effects of fibrin deposition on endothelialization, we conducted co‐culture experiments under dynamic flow conditions. The results indicated that fibrin deposition enhanced endothelialization by promoting endothelial cell adhesion and proliferation. Furthermore, we highlighted the important effects of VEGF‐A on endothelialization after iron stent corrosion. Our study as a whole is the first to describe the role of iron stent corrosion in rapid endothelialization, pointing to a new direction for enhancing endothelialization after stenting (Figure S2).

## 5 METHODS AND MATERIALS

### 5.1 Device parameters and performance

The detailed microstructure and composition of nitrided iron stents (Fe alloyed with 0.074 wt% N) used in this study have been previously described.^47^ Nitrided iron stents and 316L type stainless steel stent (S316L stent) had identical designs, and the parameters of the two stents were as follows: Φ3.0 × 18 mm, thickness 90 μm. Both the nitrided iron and S316L stent were manufactured by Lifetech Scientific Co., Ltd. (Shenzhen, China). A nitrided iron stent was obtained by vacuum plasma nitriding at 500°C and 50 Pa pressure (N_2_:H_2_ = 1:3) for 2 h. The stents were electrochemically polished and then crimped onto the inflatable balloon of a rapid exchange catheter (Φ3.0 × 18 mm) using an automatic crimping machine. All instruments were sterilized using ethylene oxide. The iron stent was corroded by placing it in HCl (3 mmol/L) for 2 h, after which the debris was polished with tartaric acid.

### 5.2 Iron stent corrosion

As shown in Figure 1, the experimental device consisted of three different parts: the pumping system, monitoring system, and conduit‐connected setup. First, one end of the silastic conduit was connected to the recycling pump, and the other end was placed into a reservoir filled with plasma. The pump was then immersed in the reservoir, and the plasma was transferred from one end to the other. The monitoring system was added to the conduit system and used to measure experimental parameters, such as temperature, pH, and flow dynamic velocity. The temperature was set between 36 and 37°C, and the pH ranged from 7.35 to 7.45. A new iron stent was deployed in the middle of the conduit, and another S316L stent was used as a control. Stent appearances and corrosion pits were observed during prolonged corrosion. The experiment was performed in triplicate.

### 5.3 Rabbit arteriovenous shunt

Ex vivo arteriovenous shunt models in rabbits were used to evaluate the adhesion of blood components to the corroded iron stents. The initial mass of the stent was recorded before implantation. On the day before the arteriovenous shunt experiment, the animals received a loading dose of 25 mg of clopidogrel and aspirin to prevent platelet accumulation. All rabbits received diazepam (1 mg/kg) and ketamine (25 mg/kg) via subcutaneous injection for anesthesia with mechanical ventilation. The right femoral artery was surgically exposed, and a 4F guide catheter was introduced over a 0.356 mm guidewire. The right femoral vein was then exposed, and a transparent silicone conduit was immediately connected to the guide catheter and vein. Two corroded iron stents (Φ3.0 × 18 mm, thickness 90 μm) were placed in the middle of the conduit. Additionally, two new iron stents without corrosion (Φ3.0 × 18 mm, thickness 90 μm) were used as the control and deployed close to the corroded stent. Seven adult rabbits were used for the experiment (weight, 5.0 kg ± 0.3 kg; 4 males and 3 females). Detailed information regarding the device is presented in Figure 2a,b.

### 5.4 Stent implantation

The corroded iron stent (Φ3.0 × 18 mm, thickness 90 μm) and the S316L stent (Φ3.0 × 18 mm, thickness 90 μm) were placed into the right carotid artery. In addition, the corroded stent covered with fibrin and the S316L stent (Φ3.0 × 18 mm, thickness 90 μm) were separately placed in the iliac artery bifurcation; 14 adult New Zealand white rabbits were included in the study (weight, 5.0 kg ± 0.4 kg; 7 males and 7 females). The initial mass of the stent was recorded before implantation. All rabbits received 25 mg of clopidogrel and 25 mg of aspirin to avoid platelet accumulation the day before implantation. All rabbits received diazepam (1 mg/kg) and ketamine (25 mg/kg) via subcutaneous injection for anesthesia with mechanical ventilation. The right femoral artery was surgically exposed, and a 4F guide catheter was introduced over a 0.356 mm guidewire. The stent was then introduced using a guidewire and deployed into the right carotid artery or the iliac artery bifurcation. Heparin (200 IU/kg) was administered to all rabbits via a catheter to maintain an activated coagulation time of >300 s during surgery. All rabbits were euthanized by narcotization and the injection of a saturated potassium chloride solution. The use of all experimental rabbits followed the accepted institutional policies, with New Zealand rabbits being approved by the Ethics Committee of the Shenzhen Testing Centre of Medical Devices. All animal experiments were approved by the Institutional Animal Ethics Committee of Xiangya Hospital (Animal Protocol Number: 2019101091).

### 5.5 Histopathology

The spleen, lung, kidney, and explanted scaffolded artery tissues were collected. To better observe tissue response, the tissue was placed in a solution containing 5 ml glacial acetic acid (99.5%), 5 ml concentrated nitric acid (65%), 10 ml formaldehyde (40%), and 80 ml ethanol (100%), for 4 h at 37°C. All samples were dehydrated with ethanol (100%) for 1 h, vitrified in xylene for 30 min, and embedded in paraffin. Paraffin‐embedded samples (4–5 μm) were prepared using a rotary microtome (LEICA RM2235; Germany). All slides underwent xylene dewaxing, gradient rehydration, and neutralization for 6 min in a 0.05 M sodium hydroxide solution, followed by rinsing and hematoxylin and eosin staining. Histopathological evaluation was performed using an optical microscope (Leica DM6000 B; Solms, Germany).

### 5.6 Scanning transmission electron microscopy

All samples obtained from the vasculature surrounding the implanted prosthesis were processed by sectioning (50 nm) polished cross sections, and the images were captured with a Leica EM UC6 ultramicrotome. Briefly, images of stented arteries were obtained. The embedded tissue samples were then cross‐sectioned into 1.5 mm‐thick slices and successively ground with 1200, 2000, and 7000 grit silicon carbide grinding papers. Fibrin deposition on the stent surface was observed using STEM (JSM‐6510; JEOL, Japan). Three images were acquired for each sample.

### 5.7 Artery angiography

Carotid and iliac artery angiography images were obtained using the CGO‐2100 Cath‐Lab system (Wandong, China). An angiographic catheter is inserted proximally to the target artery. The contrast agent was injected into the target artery using an angiographic catheter. X‐ray images of the same location before and after contrast agent injection were collected using a CGO‐2100 Cath‐Lab system. The subtracted images were immediately processed using a CGO‐2100 Cath‐Lab system.

### 5.8 Optical coherence tomography

The C7 XR Fourier‐Domain System (LightLab Imaging, Westford, MA, USA) was used to collect OCT images. First, the OCT catheter was placed in the targeted stenting segments to capture the images. A contrast reagent (iodixanol 370; GE Healthcare, Viviparus) was injected via the guiding catheter. Subsequently, the detected fiber was drawn back into the arterial lumen at a speed of 20 mm/s. The generation rate of OCT images was 100 fps.

### 5.9 Cell cycle analysis

Flow cytometry was performed to measure the cell cycle progression. Endothelial cells used for flow cytometry were obtained from rabbit vascular tissue that was serum‐starved overnight. The cells were then stimulated with platelet‐derived growth factor‐BB (PDGF‐BB) (20 ng/ml) for 20 h. Ethanol (70%) was used to fix the trypsin‐harvested cells, and the fixed cells were stored at 4°C. The cells were resuspended in phosphate‐buffered saline three times. Subsequently, all cells were incubated with a propidium iodide solution (50 μg/ml, 400 μl, and 100 μg/ml RNase A). Fluorescence was detected and measured using a FACSCalibur flow cytometer (Becton Dickinson). The percentage of endothelial cells in the G0/G1, S, and G2/M phases was calculated. The experiments were performed in triplicate.

### 5.10 Statistical analysis

SPSS version 18.0 (SPSS Inc., Chicago, USA) was used for the statistical analysis. A one‐way analysis of variance was used to assess the statistical differences between groups. Statistical significance was set at a *p*‐value <0.05.

## AUTHOR CONTRIBUTIONS

**Yalan Deng:** Conceptualization (equal); data curation (equal); formal analysis (lead); investigation (equal); methodology (equal); project administration (equal); software (equal); writing – original draft (lead). **Yanbin Wen:** Conceptualization (equal); data curation (equal); methodology (equal); supervision (equal); visualization (equal). **Jun Yin:** Data curation (equal); project administration (equal); software (equal); supervision (equal); validation (equal); visualization (equal). **Jiabing Huang:** Conceptualization (equal); data curation (equal); formal analysis (equal); methodology (equal); project administration (equal); visualization (equal). **Rongsen Zhang:** Data curation (equal); methodology (equal); resources (equal); software (equal); validation (equal). **Gui Zhang:** Conceptualization (equal); methodology (equal); project administration (equal); resources (equal); software (equal); supervision (equal); visualization (equal). **Dongxu Qiu:** Conceptualization (equal); data curation (equal); funding acquisition (lead); investigation (lead); resources (lead); supervision (equal); writing – review and editing (lead).

## CONFLICT OF INTEREST

The authors declare no competing interests.

### PEER REVIEW

The peer review history for this article is available at https://publons.com/publon/10.1002/btm2.10469.

## Supporting information


**Figure S1:** Supporting Information

**Figure S2:** Supporting Information

## Data Availability

The data supporting the findings of this study are available from the corresponding authors upon reasonable request.

## References

[1] Malakar AK , Choudhury D , Halder B , Paul P , Uddin A , Chakraborty S . A review on coronary artery disease, its risk factors, and therapeutics. J Cell Physiol. 2019;234(10):16812‐16823. 30790284 10.1002/jcp.28350

[2] Ullrich H , Olschewski M , Munzel T , Gori T . Coronary in‐stent restenosis: predictors and treatment. Dtsch Arztebl Int. 2021;118(38):637‐644. 34379053 10.3238/arztebl.m2021.0254 PMC8715314

[3] Geith MA , Nothdurfter L , Heiml M , et al. Quantifying stent‐induced damage in coronary arteries by investigating mechanical and structural alterations. Acta Biomater. 2020;116:285‐301. 32858190 10.1016/j.actbio.2020.08.016

[4] Ullrich H , Munzel T , Gori T . Coronary stent thrombosis‐ predictors and prevention. Dtsch Arztebl Int. 2020;117(18):320‐326. 32605709 10.3238/arztebl.2020.0320 PMC7358792

[5] Tesfamariam B . Endothelial repair and regeneration following intimal injury. J Cardiovasc Transl Res. 2016;9(2):91‐101. 26797874 10.1007/s12265-016-9677-1

[6] Hristov M , Weber C . Endothelial progenitor cells in vascular repair and remodeling. Pharmacol Res. 2008;58(2):148‐151. 18722530 10.1016/j.phrs.2008.07.008

[7] Scott NA , Candal FJ , Robinson KA , Ades EW . Seeding of intracoronary stents with immortalized human microvascular endothelial cells. Am Heart J. 1995;129:860‐866. 7732973 10.1016/0002-8703(95)90104-3

[8] Colombo A , Chieffo A , Frasheri A , et al. Second‐generation drug‐eluting stent implantation followed by 6‐ versus 12‐month dual antiplatelet therapy: the SECURITY randomized clinical trial. J Am Coll Cardiol. 2014;64(20):2086‐2097. 25236346 10.1016/j.jacc.2014.09.008

[9] Didier R , Morice MC , Barragan P , et al. 6‐ versus 24‐month dual antiplatelet therapy after implantation of drug‐eluting stents in patients nonresistant to aspirin: final results of the ITALIC trial (is there a life for DES after discontinuation of Clopidogrel). JACC Cardiovasc Interv. 2017;10(12):1202‐1210. 28641840 10.1016/j.jcin.2017.03.049

[10] Oberhauser JP , Hossainy S , Rapoza RJ . Design principles and performance of bioresorbable polymeric vascular scaffolds. EuroIntervention. 2009;5(Suppl F):F15‐F22. 22100671 10.4244/EIJV5IFA3

[11] Onuma Y , Muramatsu T , Kharlamov A , Serruys PW . Freeing the vessel from metallic cage: what can we achieve with bioresorbable vascular scaffolds? Cardiovasc Interv Ther. 2012;27(3):141‐154. 22569783 10.1007/s12928-012-0101-8

[12] Serruys PW , Garcia‐Garcia HM , Onuma Y . From metallic cages to transient bioresorbable scaffolds: change in paradigm of coronary revascularization in the upcoming decade? Eur Heart J. 2012;33(1):16‐25b. 22041548 10.1093/eurheartj/ehr384

[13] Qing W , Behnam A , Feng J , et al. Catalytic formation of nitric oxide mediated by Ti–Cu coatings provides multifunctional interfaces for cardiovascular applications. Adv Mater Interfaces. 2018;8:23.

[14] Ming Y , Yong W , Fang Y , et al. Shellac: a bioactive coating for surface engineering of cardiovascular devices. Adv Mater Interfaces. 2022;9(15):2102526.35538925

[15] Sun A , Huang X , Jiao Y , Wang X , Wen J . Construction of biological factor‐coated stent and its effect on promoting endothelialization. Mater Sci Eng C Mater Biol Appl. 2018;122:111943. 10.1016/j.msec.2021.111943 33641929

[16] Hou YC , Li JA , Zhu SJ , et al. Tailoring of cardiovascular stent material surface by immobilizing exosomes for better pro‐endothelialization function. Colloids Surf B Biointerfaces. 2020;189:110831. 32058252 10.1016/j.colsurfb.2020.110831

[17] Rajesh G , Behnam A , Matti AH , et al. HiPIMS carbon coatings show covalent protein binding that imparts enhanced hemocompatibility. Carbon. 2018;193:1‐16.

[18] Huang B , Jing F , Akhavan B , et al. Multifunctional Ti‐xCu coatings for cardiovascular interfaces: control of microstructure and surface chemistry. Mater Sci Eng C Mater Biol Appl. 2019;104:109969. 31500016 10.1016/j.msec.2019.109969

[19] Zhang Y , Wang J , Xiao J , et al. An electrospun fiber‐covered stent with programmable dual drug release for endothelialization acceleration and lumen stenosis prevention. Acta Biomater. 2019;94:295‐305. 31195144 10.1016/j.actbio.2019.06.008

[20] Dan Z , Jin L , Fang K , et al. Reveal crucial subtype of natural chondroitin sulfate on the functionalized coatings for cardiovascular implants. J Mater Sci Technol. 2021;117:158‐166.

[21] Kawamoto H , Ruparelia N , Tanaka A , Chieffo A , Latib A , Colombo A . Bioresorbable scaffolds for the management of coronary bifurcation lesions. JACC Cardiovasc Interv. 2016;9(10):989‐1000. 27198679 10.1016/j.jcin.2016.02.038

[22] Saito N , Mori Y , Komatsu T . Influence of stent flexibility on artery wall stress and wall shear stress in bifurcation lesions. Med Devices (Auckl). 2020;13:365‐375. 33173357 10.2147/MDER.S275883 PMC7646508

[23] Wekesah FM , Kyobutungi C , Grobbee DE , Klipstein‐Grobusch K . Understanding of and perceptions towards cardiovascular diseases and their risk factors: a qualitative study among residents of urban informal settings in Nairobi. BMJ Open. 2019;9(6):e026852. 10.1136/bmjopen-2018-026852 PMC6588962 31209088

[24] Writing Group M , Mozaffarian D , Benjamin EJ , et al. Executive summary: Heart Disease and Stroke Statistics‐‐2016 update: a report from the American Heart Association. Circulation. 2016;133(4):447‐454. 26811276 10.1161/CIR.0000000000000366

[25] Kruger‐Genge A , Blocki A , Franke RP , Jung F . Vascular endothelial cell biology: an update. Int J Mol Sci. 2019;20:18. 10.3390/ijms20184411 PMC6769656 31500313

[26] Neubauer K , Zieger B . Endothelial cells and coagulation. Cell Tissue Res. 2022;387(3):391‐398. 34014399 10.1007/s00441-021-03471-2 PMC8975780

[27] Regazzoli D , Leone PP , Colombo A , Latib A . New generation bioresorbable scaffold technologies: an update on novel devices and clinical results. J Thorac Dis. 2017;9(Suppl 9):S979‐S985. 28894604 10.21037/jtd.2017.07.104 PMC5583081

[28] Sotomi Y , Onuma Y , Collet C , et al. Bioresorbable scaffold: the emerging reality and future directions. Circ Res. 2017;120(8):1341‐1352. 28408454 10.1161/CIRCRESAHA.117.310275

[29] Lin W , Qin L , Qi H , et al. Long‐term in vivo corrosion behavior, biocompatibility and bioresorption mechanism of a bioresorbable nitrided iron scaffold. Acta Biomater. 2017;54:454‐468. 28315492 10.1016/j.actbio.2017.03.020

[30] Lin W , Zhang H , Zhang W , et al. In vivo degradation and endothelialization of an iron bioresorbable scaffold. Bioact Mater. 2021;6(4):1028‐1039. 33102944 10.1016/j.bioactmat.2020.09.020 PMC7566209

[31] Sun G , Guo W , Zhang H , Ma X , Jia X , Xiong J . A novel iron‐bioresorbable sirolimus‐eluting scaffold device for infrapopliteal artery disease. JACC Cardiovasc Interv. 2022;15(5):e57‐e59. 35151611 10.1016/j.jcin.2021.12.017

[32] Qiu D , Deng Y , Wen Y , et al. Iron corroded granules inhibiting vascular smooth muscle cell proliferation. Mater Today Bio. 2022;16:100420. 10.1016/j.mtbio.2022.100420 PMC9468459 36110422

[33] Kolandaivelu K , Swaminathan R , Gibson WJ , et al. Stent thrombogenicity early in high‐risk interventional settings is driven by stent design and deployment and protected by polymer‐drug coatings. Circulation. 2011;123(13):1400‐1409. 21422389 10.1161/CIRCULATIONAHA.110.003210 PMC3131199

[34] Litvinov RI , Pieters M , de Lange‐Loots Z , Weisel JW . Fibrinogen and fibrin. Subcell Biochem. 2021;96:471‐501. 33252741 10.1007/978-3-030-58971-4_15

[35] Weisel JW , Litvinov RI . Fibrin formation, structure and properties. Subcell Biochem. 2017;82:405‐456. 28101869 10.1007/978-3-319-49674-0_13 PMC5536120

[36] Weisel JW . Fibrinogen and fibrin. Adv Protein Chem. 2005;70:247‐299. 15837518 10.1016/S0065-3233(05)70008-5

[37] Calonder C , Matthew HW , Van Tassel PR . Adsorbed layers of oriented fibronectin: a strategy to control cell‐surface interactions. J Biomed Mater Res A. 2005;75(2):316‐323. 16059890 10.1002/jbm.a.30417

[38] Potts JR , Campbell ID . Structure and function of fibronectin modules. Matrix Biol. 1996;15(5):313‐320; discussion 321. 8981327 10.1016/s0945-053x(96)90133-x

[39] Filova E , Brynda E , Riedel T , et al. Improved adhesion and differentiation of endothelial cells on surface‐attached fibrin structures containing extracellular matrix proteins. J Biomed Mater Res A. 2014;102.3:698‐712. 23723042 10.1002/jbm.a.34733

[40] Carr HM , Vohra R , Sharma H , et al. Endothelial cell seeding kinetics under chronic flow in prosthetic grafts. Ann Vasc Surg. 1996;10(5):469‐475. 8905067 10.1007/BF02000595

[41] Thomson GJ , Vohra R , Walker MG . Cell seeding for small diameter ePTFE vascular grafts: a comparison between adult human endothelial and mesothelial cells. Ann Vasc Surg. 1989;3(2):140‐145. 2765355 10.1016/S0890-5096(06)62007-4

[42] Arnout J , Hoylaerts MF , Lijnen HR . Haemostasis. Handb Exp Pharmacol. 2006;176(Pt 2):1‐41. 10.1007/3-540-36028-x_1 17001771

[43] Ruggeri ZM . von Willebrand factor and fibrinogen. Curr Opin Cell Biol. 1993;5(5):898‐906. 8240833 10.1016/0955-0674(93)90041-n

[44] Ferrara N . Vascular endothelial growth factor: basic science and clinical progress. Endocr Rev. 2004;25(4):581‐611. 15294883 10.1210/er.2003-0027

[45] Hirashima M . Regulation of endothelial cell differentiation and arterial specification by VEGF and notch signaling. Anat Sci Int. 2009;84(3):95‐101. 19259767 10.1007/s12565-009-0026-1

[46] Wiszniak S , Schwarz Q . Exploring thei functions of VEGF‐A. Biomolecules. 2021;11:1. 10.3390/biom11010128 PMC7835749 33478167

[47] Lin W , Zhang G , Cao P , et al. Cytotoxicity and its test methodology for a bioabsorbable nitrided iron stent. J Biomed Mater Res B Appl Biomater. 2015;103(4):764‐776. 25065998 10.1002/jbm.b.33246

