# Supplementary figures and images for: Corroded iron stent increases fibrin deposition and promotes endothelialization after stenting
Source: Bioeng Transl Med. 2022 Dec 13;8(3):e10469. doi: 10.1002/btm2.10469 (PMC10189476; doi:10.1002/btm2.10469)

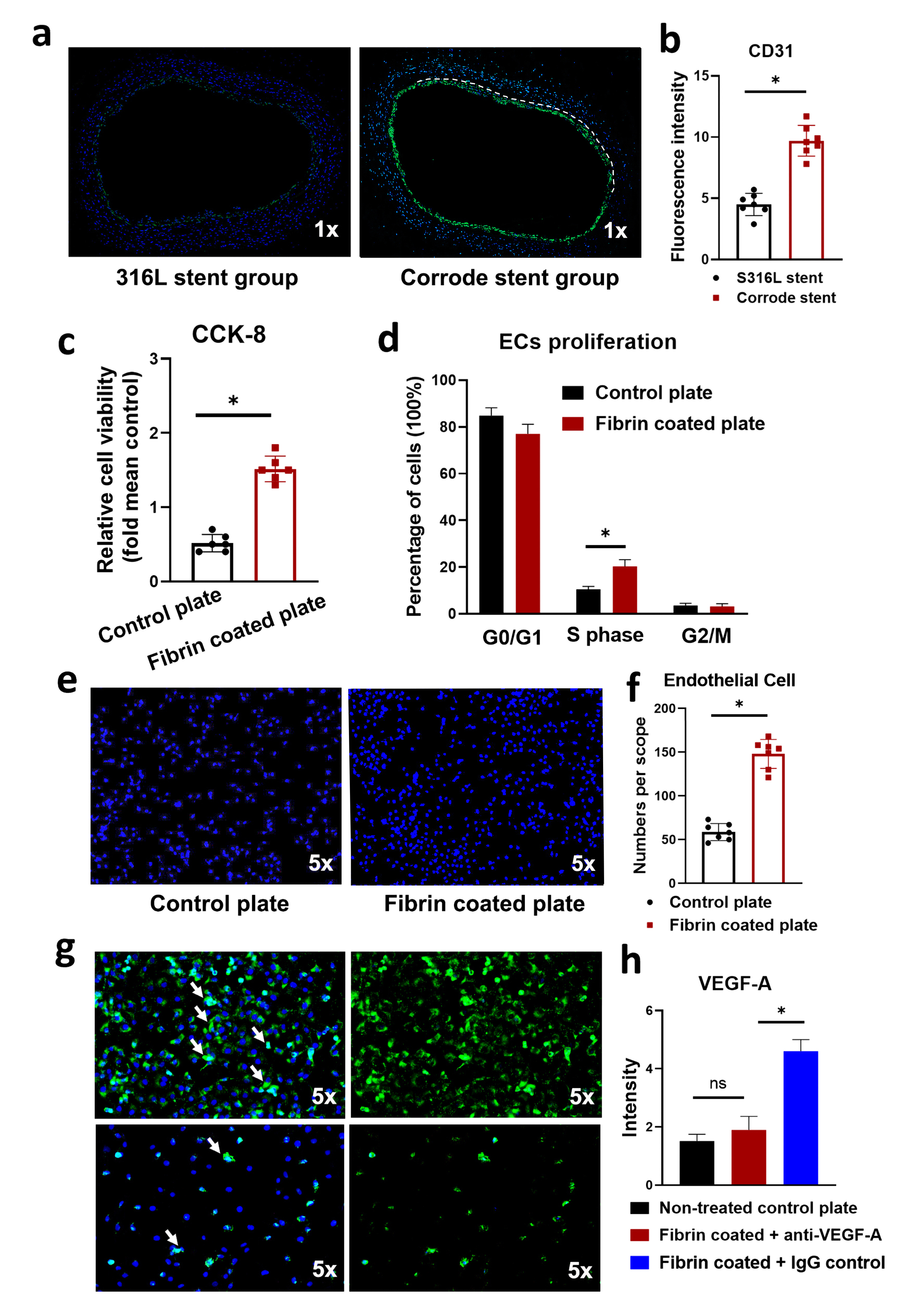

Supplement: Supplementary file 1 — Figure S1: Supporting Information [file BTM2-8-e10469-s002.jpg]

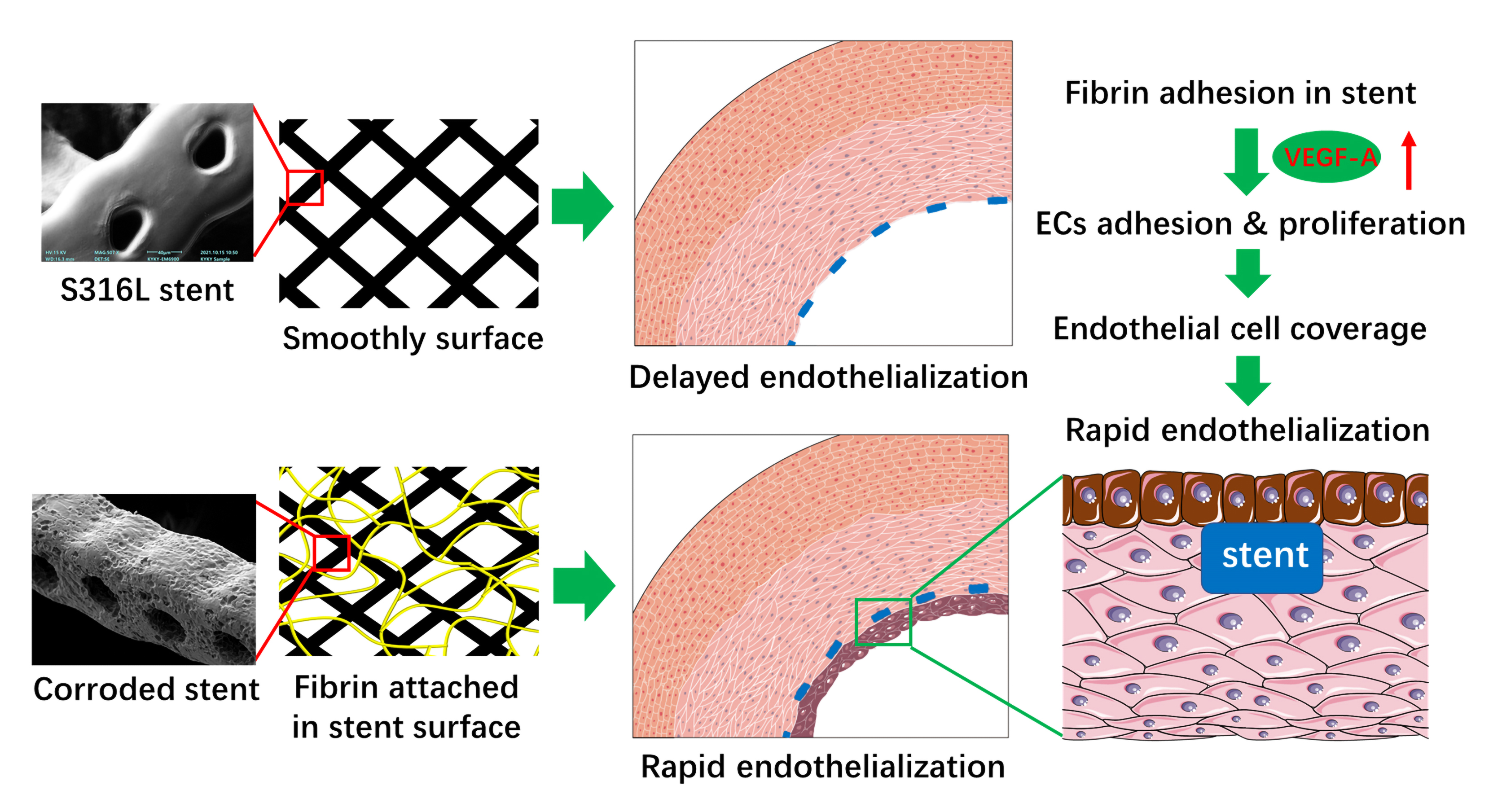

Supplement: Supplementary file 2 — Figure S2: Supporting Information [file BTM2-8-e10469-s001.jpg]
